# Screening of host proteins interacting with the African swine fever virus outer membrane protein CD2v

**DOI:** 10.3389/fmicb.2025.1585335

**Published:** 2025-06-30

**Authors:** Xiong-nan Chen, Gao-xi Zhu, Yi-xian Li, Yi-han Hao, Yi-fan Liang, Chen Hu, Ying-shuo Sun, Yun-zhao Peng, Xi Li, Zhao Huang, Gui-hong Zhang, Lang Gong, Ze-zhong Zheng

**Affiliations:** ^1^Guangdong Provincial Key Laboratory of Zoonosis Prevention and Control, College of Veterinary Medicine, South China Agricultural University, Guangzhou, China; ^2^Research Center for African Swine Fever Prevention and Control, South China Agricultural University, Guangzhou, China; ^3^Maoming Branch, Guangdong Laboratory for Lingnan Modern Agriculture, Guangdong, China; ^4^School of Medicine, Shenzhen Campus of Sun Yat-sen University, Sun Yat-sen University, Shenzhen, China; ^5^Key Laboratory of Animal Vaccine Development, Ministry of Agriculture and Rural Affairs, Guangzhou, China; ^6^Wen’s Food Group, Yunfu, China

**Keywords:** African swine fever virus, CD2v, dual membrane system, membrane protein interaction, interaction network

## Abstract

**Background:**

The African swine fever virus (ASFV) is a highly pathogenic double-stranded DNA virus that poses a significant threat to the global swine industry. Although the CD2v protein encoded by ASFV is a key factor in viral immune evasion and pathogenicity, the mechanism underlying its interaction with host proteins remains unclear.

**Methods:**

The aim of this study was to identify a range of potential host proteins that interact with the CD2v protein using the membrane yeast two-hybrid technology.

**Results:**

Through subsequent validation experiments and functional analyses, we discovered that these proteins are involved in critical cellular processes such as translational regulation, inflammatory responses, immune signaling, and iron metabolism. Furthermore, interaction network and functional enrichment analyses revealed that ASFV might influence host cell functions through multiple pathways to facilitate viral replication.

**Conclusion:**

This study provides new insights into the pathogenic mechanisms of ASFV and offers valuable clues for identifying antiviral targets.

## 1 Introduction

The African swine fever virus (ASFV) is one of the most threatening pathogens causing infectious disease and affecting the global swine industry. The complex double-stranded DNA genome encodes many proteins that play critical roles in viral infection, replication, and transmission (Alejo et al., 2018). A distinguishing characteristic of ASFV is its ability to hijack host molecular mechanisms to enhance viral growth and immune evasion. However, the molecular mechanisms by which the key envelope proteins associated with viral particles perform these complex functions remain largely unexplored.

The CD2v protein encoded by the *EP402R* gene in ASFV is a highly conserved envelope protein that performs multiple functions during the viral life cycle, including facilitating the dissemination of viral particles and suppressing the host immune response (Abkallo et al., 2022; Rodriguez et al., 1993; Song et al., 2014). CD2v is often considered a candidate protein for vaccine development (Liu et al., 2023; Song et al., 2023). In ASFV-infected cells, the expression of CD2v induces hemadsorption, during which porcine red blood cells adhere to macrophages to form rosette-like structures. This phenomenon facilitates the absorption of heme by red blood cells surrounding the macrophages and assists in the adhesion of viral particles to red blood cells, promoting viral dissemination and transmission within the host (Petrovan et al., 2022; Ruiz-Gonzalvo and Coll, 1993). In addition, the CD2v protein interacts with the actin-binding adapter protein SH3P7. It has been hypothesized that the binding of CD2v to SH3P7 is involved in protein regulation and transport to the surface of ASFV-infected cells (Kay-Jackson et al., 2004). CD2v has also been reported to interact with host CSF2RA, modulating the JAK2-STAT3 pathway to suppress apoptosis and enhance viral replication (Gao Q. et al., 2023). However, the interaction network of CD2v with other host proteins and its molecular mechanisms at the cellular level remain poorly understood and insufficient in-depth studies are available.

As CD2v is a highly conserved envelope protein, traditional yeast two-hybrid screening methods have certain limitations for membrane proteins. This study aimed to comprehensively analyze the interaction profile between the ASFV CD2v protein and host cell proteins using a membrane yeast two-hybrid screening system, the DUAL membrane system, which is specifically designed for screening interacting partners of membrane proteins. The identified interactions were validated through various experiments to confirm their authenticity and biological significance. By integrating a molecular functional network analysis and functional enrichment studies, we explored the potential roles of these host proteins in the viral infection process and their relevance to ASFV infection strategies. This research not only provides new insights into the mechanisms underlying ASFV-host interactions, but also presents an important theoretical foundation for developing antiviral therapeutic strategies based on host-virus interactions.

## 2 Results

### 2.1 Identification of 30 potential host proteins interacting with ASFV CD2v using the DUAL membrane system

**FIGURE 1:**
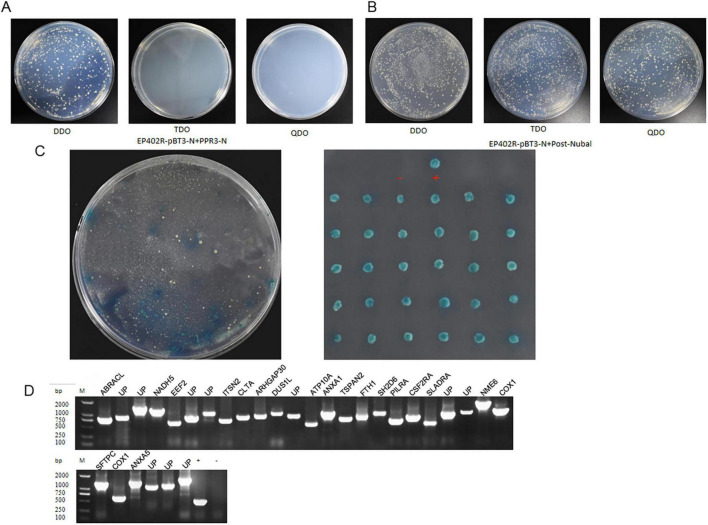
The DUAL membrane system identified 30 potential host proteins that may interact with the ASFV CD2v protein. **(A)** Yeast cells co-transformed with the bait plasmid CD2v-pBT3 and the empty prey plasmid pPR3-N were plated on DDO (SD/-Leu/-Trp)/X-α-Gal, TDO (SD/-Leu/-Trp/-His)/X-α-Gal, and QDO (SD/-Leu/-Trp/-His/-Ade)/X-α-Gal selective media. **(B)** Yeast cells co-transformed with the bait plasmid CD2v-pBT3 and the autoactivation-positive control POST-Nubal were plated on DDO, TDO, and QDO selective media. **(C)** Yeast cells co-transformed with the bait plasmid CD2v-pBT3 and prey host protein plasmids pPR3-N were plated on TDO selective media, and blue colonies were subsequently plated on QDO selective media. “+” indicates a positive interaction control (co-transformation of PTSU2-APP and PNubG-Fe65), whereas “–” represents a negative interaction control (co-transformation of PTSU2-APP and empty pPR3-N). **(D)** PCR validation was performed using universal primers for the prey plasmids. Lanes 1–30 represent PCR products from positive clones. Lane “+” is the positive control (PCR using pPR3-N as the template), lane “–” is the negative control, and Lane “UP” is Uncharacterized protein, (PCR using water as the template and all host protein name abbreviations can be found in Supplementary Table S2).

To identify the host proteins interacting with ASFV CD2v, we constructed and transformed the yeast bait plasmid CD2v-pBT3 and the prey plasmid pPR3-N into NMY51 yeast cells. The results showed that the plasmids allowed yeast growth on DDO (SD/-Leu/-Trp)/X-α-Gal plates but not on TDO (SD/-Leu/-Trp/-His)/X-α-Gal or QDO (SD/-Leu/-Trp/-His/-Ade)/X-α-Gal plates (Figure 1A). This indicated that the recombinant CD2v-pBT3 bait plasmid was not toxic to the host yeast strain and did not autoactivate the reporter genes *His3* and *ADE2*. Subsequently, we co-transformed the autoactivation-positive control POST-Nubal with the bait plasmid into NMY51 yeast cells, and observed that the co-transformed yeast grew on DDO, TDO, and QDO media (Figure 1B). This confirmed successful co-transformation and demonstrated that the CD2v fusion protein retained its normal function, allowing the ubiquitin system to activate the reporter genes *His3* and *ADE2*, making it suitable for subsequent screening experiments.

Next, the bait plasmid CD2v-pBT3 was transformed into the NMY51 yeast strain to generate a bait strain. Using a porcine alveolar macrophage (PAM) yeast two-hybrid cDNA library constructed in our previous study, we transformed the library ligation products into a bait strain and conducted two rounds of hybrid screening. During screening, we selected blue-positive colonies that grew on both TDO and QDO media (Figures 1B,C). For the positive clones, PCR validation was performed using universal primers, and plasmids from the positive clones were extracted for sequencing analysis (Figure 1D). We identified 30 host proteins that could potentially interact with CD2v.

### 2.2 ASFV CD2v co-localizes with host proteins EEF2, ANXA1, ANXA5, PILRA, SLADRA, CLTA, and FTH1

**FIGURE 2:**
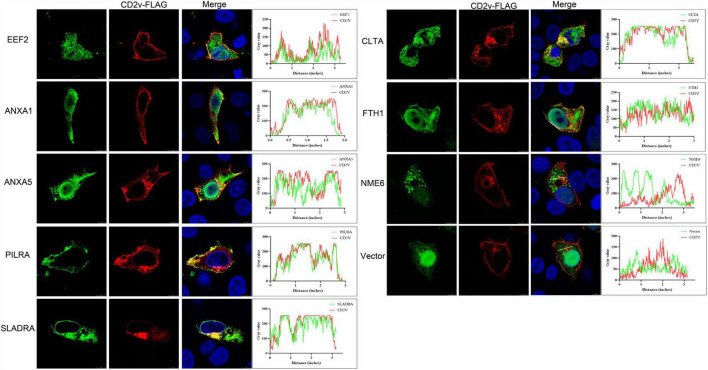
Co-localization of ASFV CD2v and host proteins. **(A)** WSL cells were transfected with ASFV CD2v-FLAG (1.25 μg) and host proteins (EEF2, ANXA1, ANXA5, PILRA, SLADRA, CLTA, FTH1, and NME6), HA (1.25 μg), or empty vector control (2.5 μg) plasmids for 24 h, and then the cells were fixed with 4% PFA for immunofluorescence. Immunofluorescence confocal microscopy shows colocalization of the FLAG-tag protein (red) and HA-tag protein (green). Scale bars, 5 μm.

To validate the interacting proteins identified through yeast two-hybrid screening, we selected eight proteins of interest: EEF2, ANXA1, ANXA5, PILRA, SLADRA, CLTA, ferritin heavy chain 1 (FTH1), and NME6. These proteins are involved in various biological processes, including metabolism, immune regulation, signal transduction, and cellular homeostasis, and may play critical roles in pathophysiological processes. We cloned these host proteins individually into the pCAGGS-HA eukaryotic expression vector, and the ASFV CD2v protein into the pCAGGS-FLAG eukaryotic expression vector. Subsequently, we co-transfected each host protein expression plasmid with the pCAGGS-CD2v-FLAG plasmid into WSL cells. Confocal microscopy revealed that, except for NME6, which showed only minimal co-localization, EEF2, ANXA1, ANXA5, PILRA, SLADRA, CLTA, and FTH1 demonstrated significant co-localization with CD2v protein (Figure 2). These findings suggest that EEF2, ANXA1, ANXA5, PILRA, SLADRA, CLTA, FTH1, and NME6 may interact with ASFV CD2v, resulting in the consistent subcellular localization of these proteins.

### 2.3 ASFV CD2v interacts with host proteins EEF2, ANXA1, ANXA5, PILRA, SLADRA, and FTH1

**FIGURE 3:**
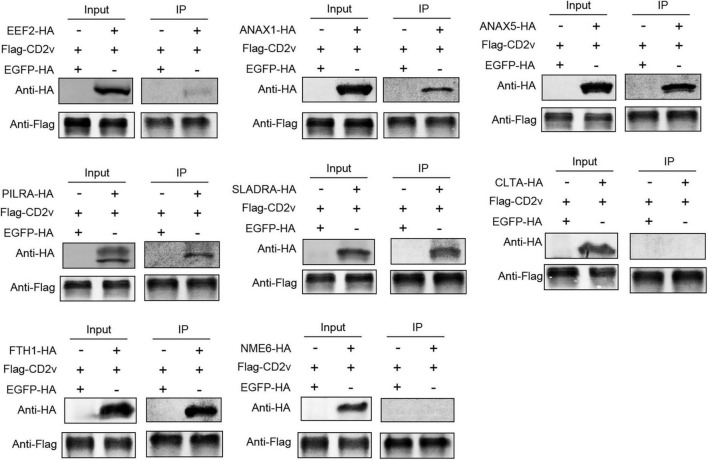
ASFV CD2v interacts with host proteins. HEK-293T cells were transfected with CD2v-FLAG (2.5 μg) and host proteins (EEF2, ANXA1, ANXA5, PILRA, SLADRA, CLTA, FTH1, and NME6), HA (2.5 μg), or empty vector control (2.5 μg) plasmids for 24 h, followed by co-IP with anti-FLAG magnetic beads and immunoblotting with anti-HA and FLAG antibody.

To investigate whether the proteins that exhibited consistent subcellular localization with CD2v also interact with it, we co-transfected HEK-293T cells with pCAGGS-HA plasmids expressing EEF2, ANXA1, ANXA5, PILRA, SLADRA, CLTA, FTH1, and NME6, along with the pCAGGS-FLAG plasmid expressing CD2v. Using co-immunoprecipitation (co-IP), we found that EEF2, ANXA1, ANXA5, PILRA, SLADRA, and FTH1 interacted with CD2v, whereas no significant interactions were detected between CD2v and CLTA or NME6 (Figure 3). These findings suggest that EEF2, ANXA1, ANXA5, PILRA, SLADRA, and FTH1 interact with CD2v, and may serve as critical factors for its functional activity.

### 2.4 Interaction network between ASFV CD2v and host proteins EEF2, ANXA1, ANXA5, PILRA, SLADRA, CLTA, FTH1, and NME6

**FIGURE 4:**
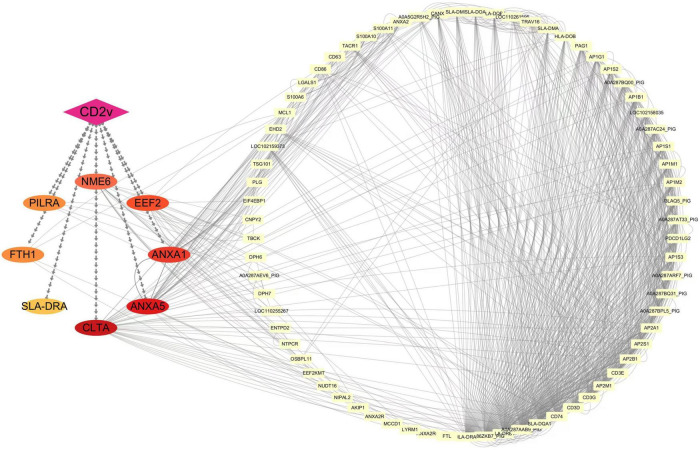
Interaction network of ASFV CD2v with host proteins. Protein-protein interactions were analyzed using the STRING database with an interaction score threshold of > 0.4. A total of 48 host proteins were selected to construct the interaction network. Each node represents a protein: on the left are proteins validated in this study interacting with ASFV CD2v, whereas on the right are additional host proteins interacting with the validated ones.

To explore the potential functional roles of these host proteins in conjunction with viral CD2v, we used Cytoscape to construct an interaction network between ASFV CD2v and the host proteins. As shown in Figure 4, the purple diamonds represent ASFV CD2v, whereas the differently colored ovals represent the eight host proteins. The yellow rectangles on the right side of the diagram indicate additional host proteins that interact with the eight host proteins. Analysis of this interaction network suggested that ASFV CD2v plays a significant role in cellular metabolism, immune regulation, signal transduction, and cellular homeostasis.

### 2.5 Gene ontology and the Kyoto encyclopedia of genes and genomes enrichment analysis of ASFV CD2v interacting with host proteins EEF2, ANXA1, ANXA5, PILRA, SLADRA, CLTA, FTH1, and NME6

To understand the role of ASFV CD2v in host cells and the biological functions of their interacting proteins, we performed GO functional enrichment and KEGG pathway enrichment analyses of the eight interacting proteins and other host proteins in the Cytoscape network.

**FIGURE 5:**
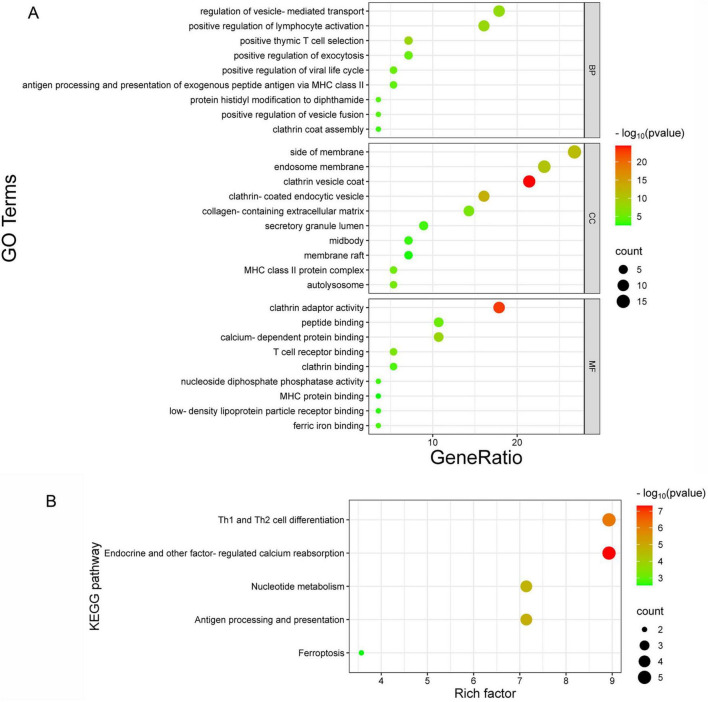
GO and KEGG pathway enrichment analysis. **(A)** GO enrichment analysis of biological processes (BP), cellular components (CC), and molecular functions (MF). The x-axis represents the gene ratio, and the y-axis shows the GO terms. **(B)** KEGG pathway enrichment analysis, where the x-axis indicates the enrichment factor, and the y-axis represents the metabolic signaling pathways.

GO functional enrichment results showed that the biological processes of these proteins primarily include immune-related functions such as antigen presentation, lymphocyte activation, and T cell selection. They are also involved in vesicle transport and secretion processes, including vesicle-mediated transport, exocytosis, vesicle fusion, and clathrin coat assembly. In addition, protein modification processes, specifically the histidine modification of diphthamide, were identified. For cellular components, enriched functions included membrane-associated structures such as endosomal membranes, membrane regions, and lipid rafts. Vesicles and endocytosis-related structures, such as clathrin-coated and endocytic vesicles, were also enriched. Specific organelles, including MHC class II complexes and autolysosomes, as well as specialized cell regions, such as the secretory granule lumen and midbody, are highlighted. Extracellular structures, such as the extracellular matrix containing collagen, were also identified. For molecular function, the analysis revealed binding-related functions, such as binding to specific molecules, including peptides, calcium ions, T cell receptors, clathrin, MHC proteins, lipoprotein receptors, and iron ions, which are crucial for molecular recognition and regulation. Enzymatic activities, such as nucleoside diphosphate phosphatase activity, which participates in cellular metabolism and energy conversion, were also enriched. Additionally, protein adapter activities, such as clathrin adapter activity, were involved in vesicle transport and membrane protein regulation (Figure 5A). KEGG pathway enrichment analysis showed that the enriched pathways included Th1 and Th2 cell differentiation, which are related to immune cell functions and regulation of immune responses. Other enriched pathways include calcium reabsorption regulated by endocrine and other factors, which involve the absorption and regulation of calcium ions in the kidney and endocrine system, as well as nucleotide metabolism, which is related to fundamental biological processes, such as DNA/RNA synthesis and energy metabolism. Antigen processing and presentation mediated by MHC molecules for antigen recognition and core immune functions were also identified. Furthermore, ferroptosis, a programmed cell death process characterized by iron-dependent lipid peroxidation, was highlighted (Figure 5B). These results suggest that the ASFV CD2v protein regulates host cellular processes through multiple pathways, facilitating viral replication and proliferation. This provides further insights into the interaction mechanisms between ASFV CD2v and host proteins and highlights their potential roles in manipulating host cell biology for viral benefits.

### 2.6 ASFV infection reduces cellular sensitivity to ferroptosis

**FIGURE 6:**
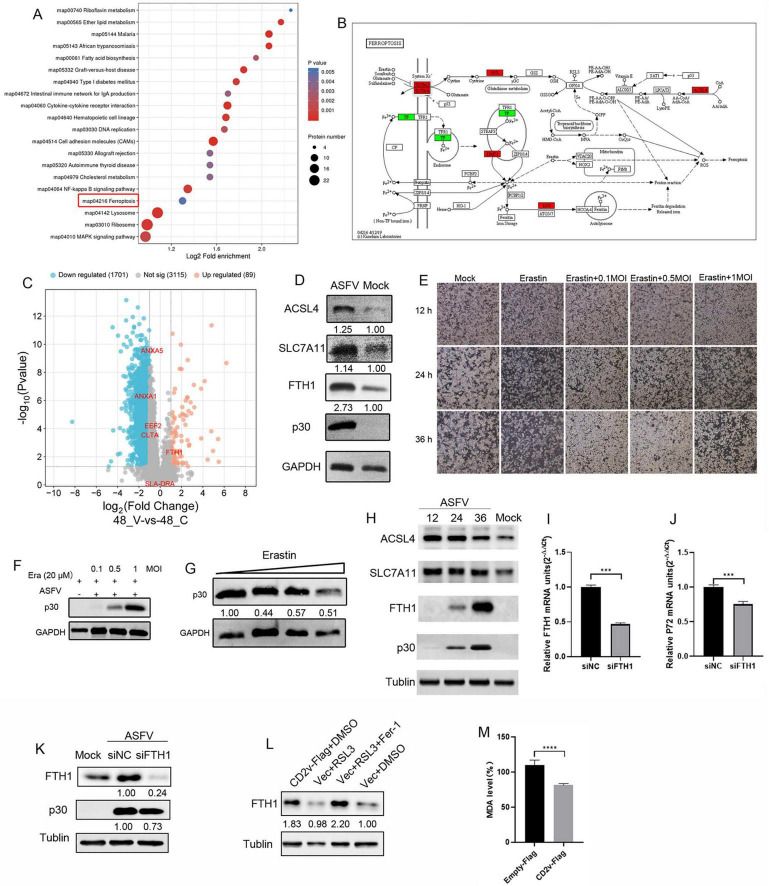
ASFV infection regulates cellular sensitivity to ferroptosis. **(A)** Bubble chart of pathway analysis from 4D proteomics in ASFV-infected cells. Each bubble represents a pathway (top 20 by *p*-value), with size indicating the number of differential proteins. The x-axis shows pathway impact, and the y-axis shows *p*-value. **(B)** Differential proteins in the ferroptosis pathway, with red indicating upregulation and green indicating downregulation. **(C)** Volcano plot of differentially expressed proteins, highlighting six validated host proteins (EEF2, ANAX1, ANAX5, SLADRA, CLTA, FTH1). **(D)** ACSL4, SLC7A11, FTH1, and p30 proteins expression in ASFV-infected (MOI = 1) PAM cells at 24 hpi. GAPDH serves as control. **(E)**, WSL cells were inoculated with ASFV (0.1, 0.5, and 1 MOI, 2 h) and incubated in maintenance medium containing erastin for 12, 24, and 36 hpi, cell death was observed under bright-field microscopy **(F)** ASFV p30 protein expression in ASFV-infected (0.1, 0.5, and 1 MOI) PAM cells at 36 hpi. GAPDH serves as control. **(G)** PAM cells were inoculated with ASFV (0.1 MOI, 2 h) and incubated in erastin-containing maintenance medium (1, 2.5, 5, and 10 μM), ASFV p30 protein expression levels were determined through Western blot at 36 hpi. **(H)** WSL cells were inoculated with ASFV MOI = 1, 2 h) for 12, 24, and 36 hpi. Western blotting of ACSL4, SLC7A11, FTH1, and p30 proteins expression levels were determined through Western blot; α-tublin served as control. **(I,J)** PAM cells were transfected with FTH1 protein-targeting siRNAs or negative control (NC) siRNA (siNC) for 48 h, followed by infection with ASFV (0.1 MOI). mRNA expression of FTH1 and viral P72 protein was determined by qRT-PCR at 24 hpi. **(K)** Western blotting of FTH1 and p30 proteins from ASFV-infected (1 MOI) siFTH1 and siNC PAM cells at 24 hpi; α-tublin serves as control. **(L)** WSL cells were transfected with CD2v-Flag plasmids or empty vector control for 24 h and treated with RSL3 (10 μM, a ferroptosis activator) or RSL3 + Ferrostatin-1 (10 μM, a ferroptosis inhibitor) or DMSO for 12 h. **(M)** WSL cells were transfected with CD2v-Flag plasmids or an empty vector control for 24 h. MDA concentrations in the cell lysates were measured according to the manufacturer’s instructions of the kit.

To analyze the role of the interacting proteins during viral infection, we examined previously published proteomic databases and found that EEF2, ANXA1, ANXA5, SLADRA, CLTA, and FTH1 were significantly enriched and FTH1 was notably upregulated in ASFV-infected PAMs (Figure 6A). Further 4D proteomic analysis of ASFV-infected PAMs revealed that in addition to the apoptosis pathway reported in earlier studies, another form of programmed cell death, ferroptosis, was significantly enriched. We identified ferroptosis-associated proteins that were significantly upregulated in this pathway (Figures 6B,C). To validate the proteomics results, we conducted ASFV infection experiments using PAMs. The results showed that ASFV infection significantly increased the expression of ACSL4, SLC7A11, and FTH1, key proteins involved in the ferroptosis pathway (Figure 6D). Among them, ACSL4 promotes ferroptosis by increasing the synthesis of polyunsaturated fatty acids, which leads to lipid peroxidation. SLC7A11 participates in antioxidant reactions by promoting the synthesis of glutathione, inhibiting the occurrence of ferroptosis. The FTH1 protein facilitates the oxidation of ferrous iron (Fe^2+^) to ferric iron (Fe^3+^), which is then stored in the ferritin complex. This process helps prevent the formation of harmful free radicals, maintain cellular iron balance, and inhibit ferroptosis (Jiang et al., 2021). To investigate the role of ferroptosis in ASFV infection, we treated the cells with the ferroptosis activator erastin after ASFV infection. The results indicated that ASFV infection significantly reduced cell death compared to that of the control group. As the multiplicity of infection (MOI) increased, the number of dead cells gradually increased, but remained lower than that of the control group (Figure 6E). Immunoblotting analysis showed that erastin treatment led to a significant reduction in glyceraldehyde-3-phosphate dehydrogenase (GAPDH) abundance, whereas ASFV infection rescued this reduction. However, increasing the ASFV MOI did not further increase GAPDH abundance (Figure 6F). To explore whether ASFV suppresses ferroptosis to promote viral replication, we treated ASFV-infected PAMs with erastin. The results showed that as the erastin concentration increased, ASFV p30 expression decreased, although this change did not show a clear dose-dependent effect (Figure 6G). These findings suggest that ASFV reduces the cellular sensitivity to ferroptosis. To investigate the regulation of ferroptosis pathways by ASFV in other susceptible cell types, we performed infection experiments in another ASFV-susceptible cell line, WSL. The results showed that ASFV infection upregulated the expression of ACSL4, SLC7A11, and FTH1. However, the abundance of ACSL4 and SLC7A11 gradually decreased over time, whereas FTH1 protein abundance continued to increase (Figure 6H). To further investigate the role of FTH1 in ASFV replication, specific siRNA was used to knock down FTH1 protein in PAMs, and ASFV replication levels were assessed. The results showed that as FTH1 mRNA levels decreased, the ASFV p72 mRNA expression level significantly reduced (Figures 6I,J). Immunoblotting results indicated that with the knockdown of FTH1 expression, ASFV p30 expression levels also decreased (Figure 6K). To further clarify the relationship between ASFV CD2v and FTH1, CD2v was overexpressed in WSL cells to observe its effect on FTH1 expression levels. The results showed that, compared to the empty vector control, overexpression of CD2v upregulated FTH1 expression levels (Figure 6L). On the other hand, compared to the empty vector control, overexpression of CD2v significantly reduced the levels of the lipid peroxidation marker malondialdehyde (MDA) in the cells (Figure 6M). These findings suggest that ASFV may promote its replication by modulating the ferroptosis pathway and that this regulation occurs in different susceptible cell lines.

## 3 Discussion

ASFV has recently caused significant economic losses to the global swine industry. The ASFV complex genome and its encoded proteins play critical roles in the viral life cycle and pathogenesis. This study, using membrane yeast two-hybrid technology combined with multiple validation experiments, identified several host interaction proteins of the ASFV envelope protein CD2v, including eukaryotic elongation factor 2 (EEF2), ANXA1, ANXA5, PILRA, SLADRA, and FTH1. Functional enrichment and proteomic analyses revealed that these host proteins highlight ASFV strategies for enhancing viral replication by modulating various host mechanisms, including translation, immune evasion, membrane dynamics, endocytosis, and iron metabolism.

EEF2 is a key ribosomal translocation factor in translational elongation (Montanaro et al., 1976). Previous studies have shown that ASFV regulates translation not only through initiation factors, such as EIF4E and EIF4G, but also through elongation factors, such as EEF2 and ribosomes, optimizing viral protein synthesis while inhibiting host protein expression (Castello et al., 2009). These factors and ribosomes are recruited to viral factories. In this study, EEF2 interacted significantly with CD2v, supporting the hypothesis that ASFV hijacks the host translation mechanisms. ASFV likely hijacks host protein synthesis through the interaction between its envelope protein and EEF2, optimizing viral protein translation, while suppressing host protein synthesis to ensure efficient replication. This mechanism is consistent with translational hijacking observed in other viruses, such as poliovirus (Lloyd, 2006).

Regarding membrane dynamics and inflammation regulation, ANXA1 and ANXA5 are membrane-associated phospholipid-binding proteins involved in inflammation and membrane repair (Gerke et al., 2005). ANXA1 plays an essential role in balancing inflammation and inhibiting mast cell activation and is a key regulatory factor in cytokine storms and vascular lesions during dengue virus infection (Costa et al., 2022). ANXA1 has also been reported to enhance viral infectivity by binding to HSV glycoprotein E (Wang et al., 2022). In this study, the interaction between ANXA1 and CD2v suggests that ASFV may evade the host immune system by regulating anti-inflammatory signaling pathways. Meanwhile, ANXA5 is critical for membrane repair and vesicle transport and has been identified as a component of influenza virus particles that inhibits interferon-gamma signaling and promote viral replication (Berri et al., 2014). Recent studies have shown that ANXA5 significantly inhibits ASFV propagation through apoptotic bodies by blocking the “eat me” signal on apoptotic bodies (Gao P. et al., 2023). This study suggests that ANXA5 supports ASFV by interfering with host membrane-related functions, aiding in viral assembly and immune evasion.

This study showed that the expression of CD2v alone can enhance the expression level of interferon IFN-β in WSL cells, while inhibiting the expression levels of pro-inflammatory factors TNF-α and IL-6 (Supplementary Figure S1), suggesting that CD2v may play an important role in the regulation of innate immunity. The paired immunoglobulin-like receptor alpha (PILRA) is an inhibitory receptor that regulates immune responses through interactions with sialic acid ligands (Fournier et al., 2000; Sun et al., 2012). Similar to its role in HSV-1, where PILRA acts as a receptor for the viral glycoprotein gB (Chowdhury et al., 2013; Satoh et al., 2008), ASFV CD2v is a transmembrane glycoprotein (Goatley and Dixon, 2011; Rodriguez et al., 1993). This study found that PILRA interacts with ASFV CD2v, suggesting that ASFV may exploit the PILRA inhibitory signaling to dampen host antiviral immune responses, creating a more favorable environment for viral replication. In addition, a member of the signaling lymphocyte activation molecule family (SLADRA) plays a vital role in immune cell activation and antigen presentation (Ladowski et al., 2018). Studies suggest that viruses can regulate host innate immunity by blocking the activation of SLA-DR α and β promoters through envelope proteins (Wang et al., 2021) or by enhancing SLA-DR-mediated viral protein antigen presentation to trigger non-neutralizing antibody responses (Wu et al., 2020). The interaction between SLADRA and ASFV CD2v suggests that ASFV modulates host immune responses by affecting T-cell or macrophage activity. Previous studies have shown that highly virulent ASFV strains can significantly impair the responsiveness of macrophages to stimuli (such as TLR ligands), manifested by reduced secretion of TNF-α, IL-6, and other cytokines (Franzoni et al., 2020). This suggests that CD2v’s ability to suppress the expression levels of pro-inflammatory factors TNF-α and IL-6 may be related to its interactions with PILRA and SLADRA, potentially contributing to the impaired macrophage immune function observed during ASFV infection. However, further research is needed to confirm this.

In terms of endocytosis and viral invasion, the clathrin light chain (CLTA) is a critical factor in clathrin-mediated endocytosis (Alkafaas et al., 2023; McMahon and Boucrot, 2011). Several studies indicate that ASFV enters cells through clathrin-mediated endocytosis (Chen X. et al., 2023; Hernaez et al., 2016). Although we did not detect a direct interaction between CD2v and CLTA, there is consistent subcellular localization of CD2v and CLTA. Since the interactions between viral proteins and host proteins may be transient or short-lived, as well as weak or of low affinity, traditional interaction detection methods, such as immunoprecipitation, are often unable to capture these brief associations (Strazic et al., 2021). For example, after rotavirus infection, several host cell ribonucleoproteins (hnRNPs) and AU-rich element-binding proteins (ARE-BPs) relocate from the nucleus to the cytoplasm and co-localize with the viral non-structural proteins NSP2 and NSP5. However, methods like immunoprecipitation have failed to detect a direct interaction between these host proteins and viral proteins (Dhillon et al., 2018). Owing to the rapid and transient nature of clathrin-mediated endocytosis signaling, the interaction between CD2v and CLTA may be difficult to detect as it occurs only for a short time. These findings suggested that ASFV CD2v may assist in triggering clathrin-mediated endocytosis to complete the invasion process, thereby providing new insights into the endocytic pathway used by ASFV to invade host cells.

Regarding iron metabolism and ferroptosis regulation, FTH1 is a key iron-storage protein that regulates intracellular iron homeostasis. Viral manipulation of FTH1 may alter cellular iron levels, favoring viral replication (Fu et al., 2024; Xu et al., 2024; Yan et al., 2023). Numerous studies have investigated ASFV regulation of cell death mechanisms. Although not yet fully elucidated, ASFV has demonstrated a remarkable ability to regulate cell death, such as by inhibiting pyroptosis by cleaving gasdermin D with the cysteine protease pS273R (Zhao et al., 2022). Recent studies have shown that the ASFV CD2v protein interacts with host CSF2RA to regulate the JAK2-STAT3 pathway and suppress apoptosis (Gao Q. et al., 2023). However, the regulation of ferroptosis by ASFV remains unclear. Recent metabolomic analyses of ASFV-infected primary PAMs revealed ferroptosis pathway enrichment at 6 hpi and an increase in arachidonic acid, a key substrate for lipid peroxidation during ferroptosis, at 12 hpi (Xue et al., 2022). Studies identified that by targeting DHODH, which block mitochondrial pyrimidine metabolism, causing lipid peroxidation, leading to ferroptosis and restricting viral replication (Chen Y. et al., 2023). In addition, ASFV enhances viral replication by increasing the intracellular levels of reduced glutathione, which plays a role in inhibiting ferroptosis (Gao et al., 2024). In this study, ASFV altered cellular iron metabolism, increasing cellular sensitivity to ferroptosis. ASFV enhanced cellular susceptibility to ferroptosis, promoting cell survival and facilitating viral replication. Although ASFV infection upregulated the expression of proteins that promote ferroptosis activation, such as ACSL4, it also upregulated the expression of proteins that inhibit ferroptosis activation, such as FTH1, ultimately reducing cellular ferroptosis. This suggests that ASFV may impede the ferroptosis process by expressing proteins like CD2v. Additionally, the study observed that viral infection increases the expression levels of ACSL4, FTH1, and SLC7A11, but they gradually decrease as the viral infection progresses. This suggests that viral infection may upregulate ACSL4, activating ferroptosis, but it also upregulates the expression of SLC7A11 and FTH1 to inhibit the ferroptosis process. This disrupts cellular iron metabolism, leading to the blockage of ferroptosis. As the viral infection progresses, the cell initiates a negative feedback mechanism to resist the virus’s control over the ferroptosis process, resulting in decreased expression of ACSL4 and SLC7A11. Notably, compared to ACSL4, the expression of SLC7A11 decreases only slightly. Furthermore, the expression of FTH1 gradually increases with viral infection. This suggests that although the cell initiates a negative feedback mechanism, the virus still inhibits the progression of ferroptosis through other pathways. In contrast, other viruses such as HSV-1 and IAV actively manipulate host proteins to induce ferroptosis, thereby promoting viral replication and enhancing their pathogenicity (Ouyang et al., 2024; Xu et al., 2023). This indicates that ASFV exhibits a unique strategy in modulating host proteins to influence ferroptosis. However, whether ASFV alters iron metabolism through CD2v-FTH1 interactions requires further investigation.

NME6 is a member of the nucleoside diphosphate kinase family that plays a central role in mitochondrial metabolism, gene expression, and protein synthesis (Mehus et al., 1999; Wanrooij and Chabes, 2023). Notably, mitochondrial networks are located at the periphery of viral factories in ASFV-infected cells (Castello et al., 2009; Netherton et al., 2004). This suggests that ASFV CD2v recruits NME6 to regulate mitochondrial function and maintain cellular homeostasis, thereby providing resources for viral DNA replication and other energy-intensive processes. Although no significant interaction was detected between NME6 and ASFV CD2v in uninfected cells, infection-induced protein modifications may alter protein localization and enhance their interaction, allowing specific biological functions.

**FIGURE 7:**
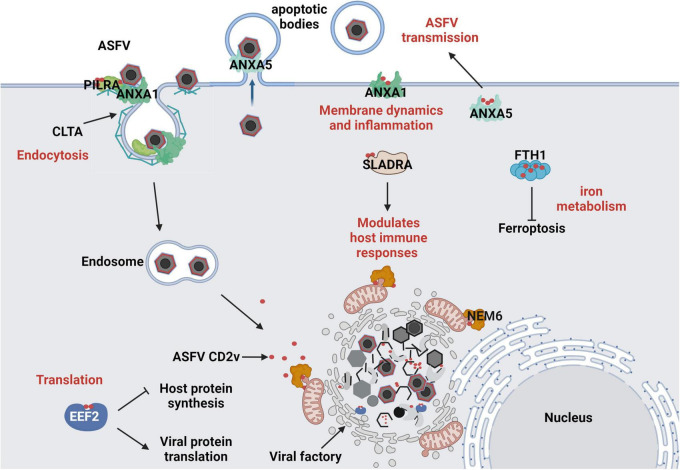
Predicted Interaction Model of ASFV CD2v with Host Cells. (1) ASFV hijacks the host translation machinery by interacting with the translation initiation factor EEF2, promoting viral protein synthesis and inhibiting host protein expression. (2) ASFV regulates membrane dynamics and inflammatory responses by interacting with ANXA1, facilitating viral entry and immune evasion. By interacting with ANXA5, it interferes with host membrane-associated functions, aiding ASFV assembly and promoting its propagation within apoptotic bodies. (3) ASFV CD2v interacts with PILRA and CLTA to promote the endocytic entry of ASFV into host cells. (4) ASFV CD2v enhances or suppresses the SLA-DR-mediated antigen presentation process of viral proteins by interacting with SLADRA, thereby modulating the host innate immune response. (5) ASFV regulates intracellular iron metabolism homeostasis through the interaction of CD2v with FTH1, promoting viral replication. (6) ASFV CD2v recruits NME6 to regulate mitochondrial function and maintain cellular homeostasis, thereby providing energy for viral DNA replication.

In conclusion, these interactions demonstrate multifaceted strategies of ASFVs in manipulating host cellular mechanisms. Targeting host factors such as EEF2 (translation), ANXA1/ANXA5 (membrane dynamics and inflammation), PILRA/SLADRA (immune regulation), CLTA (endocytosis), and FTH1 (iron metabolism) highlights the complexity of ASFV pathogenesis (Figure 7). Future studies should elucidate the molecular mechanisms underlying these interactions and their roles in ASFV virulence and immune evasion to provide new directions for antiviral strategies, including therapeutic targets and vaccine development.

## 4 Materials and methods

### 4.1 Cell culture

PAMs were prepared by lavage of the lungs of 3-week-old SPF pigs. WSL cells were kindly provided by Prof. Han Jun from China Agricultural University and maintained in RPMI 1640 medium. HEK-293T cells preserved in our laboratory were cultured in Dulbecco’s modified Eagle medium. All cells were maintained at 37°C in a humidified atmosphere with 5% CO2, using 10% fetal bovine serum (Gibco) as the culture supplement.

### 4.2 Antibodies and reagents

The antibodies against the different markers for western blotting and immunofluorescence were as follows: anti-tubulin (rabbit polyclonal, 14555-1-AP) and anti-GAPDH (mouse monoclonal antibody, clone 1E6D9) were purchased from Proteintech. HA (mouse monoclonal antibody; clone 2-2.2.14) was purchased from Invitrogen. FLAG (mouse monoclonal antibody, clone M2) was purchased from Sigma-Aldrich. Mouse monoclonal anti-p30 antibodies were generated in our laboratory. FTH1 (mouse monoclonal antibody A19544), SLC7A11 (rabbit monoclonal antibody, A2413) and ACSL4 (rabbit monoclonal antibody A20414) antibodies were purchased from Abclonal. Secondary antibodies conjugated to alexa-488, –594, or –647 were purchased from Proteintech. Goat anti-rabbit/mouse IgG H&L (HRP) was purchased from Abcam. Erastin and RSL3 was purchased from MCE. MDA Kit was purchased from Beyotime.

### 4.3 Viral stock production and titration assay

ASFV (accession no. MT496893.1) was produced in PAM cells. Briefly, PAM cells were cultured in a 100 mm cell culture dish to 100% confluence, washed one times with phosphate-buffered saline (PBS), inoculated with ASFV at an MOI of 0.1, and incubated in 1,640 with 10% fetal bovine serum at 37°C for 48 h. The culture was collected and freeze-thawed three times, centrifuged at 10,000 × g for 10 min, and the supernatant was collected and stored at –80°C.

Viral titers were determined by seeding PAM cells into 96 well plates at 100% confluence at a density of 10^5^ cells/well. Subsequently, the viral solution was diluted along a 10-fold gradient using 1,640 with 10% fetal bovine serum. Cells were prewashed one time with PBS and inoculated with 100 μL of viral solution at different dilutions. Each concentration was added to eight wells. The cells were cultured in viral maintenance medium for 48 h and HAD_50_ doses were calculated using the Reed and Muench method (Ramakrishnan, 2016).

### 4.4 Screening of the interacting proteins based on the isolated ubiquitin-mediated yeast two-hybrid system

A PAM yeast two-hybrid cDNA library was constructed as previously described. Interacting proteins were screened based on the isolated ubiquitin-mediated yeast two-hybrid system were produced as previously described. Briefly, we used the bait plasmid pBT3-N, which carries the selection marker Leu2, and the prey plasmid pPR3-N, which carries the Trp1 selection marker. The bait plasmid contained the C-terminal domain of ubiquitin fused to a transcription factor. When the N-terminal domain of ubiquitin in the prey plasmid interacts with the bait protein, complete ubiquitin is formed, activating the transcription of the reporter genes His3 and ADE2. Blue single colonies capable of growing on DDO (SD/-Leu/-Trp)/X-α-Gal, TDO (SD/-Leu/-Trp/-His)/X-α-Gal, and QDO (SD/-Leu/-Trp/-His/-Ade)/X-α-Gal plates were selected. These colonies were further analyzed using PCR and sequencing to identify the host proteins that interacted with the bait protein CD2v.

### 4.5 Plasmids and transfection

All the plasmids were constructed using the pCAGGS-HA/FLAG vector. The reference sequence for the host proteins were: EEF2, XP_020939748.1; ANXA1, NM_001163998.1; ANXA5, XP_003129266.2; PILRA, XP_020941878.1; SLA-DRA, AFN20259.1; CLTA, XP_020921680.1; FTH1, XP_005660860.1; NME6, XP_003132225.1. Target gene fragments were obtained through PCR amplification, using PAM or viral cDNA as templates.

WSL cells were seeded in in a 14-mm glass-bottom cell culture dish (Cellvis) and grown to 70–80% confluence. Plasmid transfection was performed using lipofectamine 3000. Host proteins plasmids were cotransfected with CD2v or empty plasmids (2.5 μg) for 24 h. An empty plasmid was used as a control.

The HEK-293T cells were seeded in a 100-mm cell culture dish and grown to 60–70% confluence. Plasmid transfection was performed using polyethylenimine (FUSHENBIO). Host protein plasmids were cotransfected with CD2v or empty plasmids (5 μg) for 36 h. An empty plasmid was used as a control.

PAM cells were seeded in 12-well plates with 70% confluence. Lipofectamine RNAiMAX Reagent (Invitrogen) was added with 25 nM siRNA per well for 48 h. Negative control siRNA was used as the control. All the siRNAs were purchased from RiboBio (Guangzhou, China). All primers and siRNAs targeting the gene sequences are listed in Supplementary Table S1.

### 4.6 Immunofluorescence confocal microscopy

WSL cells were cultured in a 14-mm glass-bottom cell culture dish (Cellvis). After the different experimental treatments, the cells were fixed with 4% paraformaldehyde for 15 min, washed three times with PBS at 20°C, and infiltrated with 0.3% Triton X-100 for 5 min. Cells were stained overnight with specific antibodies at 4°C or for 2 h at 37°C. The cells were washed three times with PBS and incubated with the corresponding secondary antibody at 37°C for 1 h. Cell nuclei were stained with 1 mg/mL DAPI (Beyotime).

### 4.7 Co-IP and western blotting

Cells were lysed in radioimmunoprecipitation assay lysis buffer for 30 min at 4°C. Cell lysates were collected by centrifugation at 10,000 × g and 4°C for 10 min, and proteins in the lysates were separated using sodium dodecyl-sulfate polyacrylamide gel electrophoresis (SDS-PAGE). Proteins were transferred to polyvinylidene fluoride membranes (0.45-μm pore size; Merck Millipore) and probed with the indicated antibodies (mouse anti-HA, FLAG, and rabbit anti-FLAG antibodies were purchased from Proteintech). GAPDH (Proteintech) was used as the loading control. For Co-IP, cells were lysed for 30 min at 4°C in IP Lysis/Wash Buffer (Invitrogen). Cell lysates were collected by centrifugation at 13,000 × g and 4°C for 10 min, and supernatants were incubated with specific FLAG antibodies conjugated to prewashed magnetic beads for 30 min at 25°C. Subsequently, the magnetic beads were washed with 300 μL IP Lysis/Wash Buffer and ultrapure water, collected, added to SDS-PAGE protein loading buffer, and heated at 95°C for 5 min. The supernatant was collected for western blotting and the magnetic beads were discarded. The relative protein content was calculated using ImageJ software 1.8.0.

### 4.8 Construction of a protein–protein interaction network and functional enrichment analysis

Eight validated host proteins were entered into the STRING database (version 12.0) for protein-protein interaction analysis. The interaction network was constructed using Cytoscape (version 3.10.1) with *Sus scrofa* as the target species. The interactions are represented as lines connecting the nodes. A host protein network, including proteins directly or indirectly interacting with the eight validated proteins, was constructed, annotated, and visualized using Cytoscape (version 3.8.1). The Metascape analysis website was used for GO and KEGG pathway enrichment analyses. Enriched proteomic data were further analyzed and visualized using a bioinformatics website.

### 4.9 Statistical analysis

All data, if applicable, were expressed as mean ± SD from at least three independent experiments. The Student’s *t*-test was used to compare data from pairs of treated and untreated groups. Statistical significance was set at *P* < 0.05 (^***^P < 0.001; ^**^P < 0.01; *P < 0.05; ns, *P* > 0.05). All statistical analyses and calculations were performed using Prism 8.0 software (GraphPad Software Inc., La Jolla, CA, United States).

## Data Availability

The datasets presented in this study can be found in online repositories. The names of the repository/repositories and accession number(s) can be found in the article/Supplementary material.

## Supplementary material

Supplementary Figure S1 The expression levels of antiviral cytokines IFN-β, TNF-α, and IL-6 were evaluated by qPCR. Relative expression levels of gene IFN-β, TNF-α, and IL-6 in WSL cells (in the TNF-α and IL-6 groups, WSL cells were treated with 2 μg/mL LPS for 4 h).
