# Supplementary figures and images for: Screening of host proteins interacting with the African swine fever virus outer membrane protein CD2v
Source: Front Microbiol. 2025 Jun 30;16:1585335. doi: 10.3389/fmicb.2025.1585335 (PMC12256527; doi:10.3389/fmicb.2025.1585335)

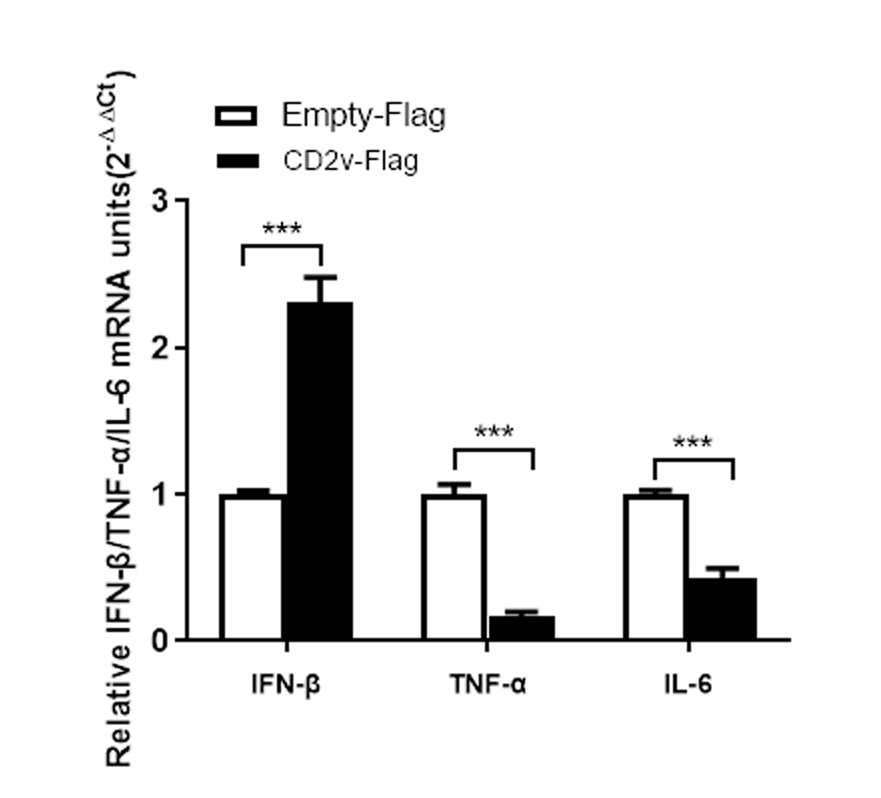

Supplement: Supplementary Figure S1 — The expression levels of antiviral cytokines IFN-β, TNF-α, and IL-6 were evaluated by qPCR. Relative expression levels of gene IFN-β, TNF-α, and IL-6 in WSL cells (in the TNF-α and IL-6 groups, WSL cells were treated with 2 μg/mL LPS for 4 h). [file Image_1.tif]
